# Incidence patterns and review of Hodgkin lymphoma in the Republic of Armenia

**DOI:** 10.3332/ecancer.2021.1319

**Published:** 2021-11-18

**Authors:** Jemma Arakelyan, Alisa Movsisyan, Lilit Sargsyan, Armine Chopikyan, Diana Andreasyan, Arevik Torosyan, Ruzanna Papyan, Hovhannes Vardevanyan, Samvel Bardakhchyan, Artashes Tadevosyan, Gevorg Tamamyan, Armen Tananyan, Samvel Danielyan, Dickran Kazandjian1,

**Affiliations:** 1 Department of Oncology, Yerevan State Medical University, 2 Koryun St, 0025 Yerevan, Armenia; 2 Department of Adult Oncology and Hematology, Hematology Center after Prof. R.H.Yeolyan, 7 Nersisyan St, 0014 Yerevan, Armenia; 3 Pediatric Cancer and Blood Disorders Center of Armenia, Hematology Center after Prof. R.H. Yeolyan, 7 Nersisyan St., 0014 Yerevan, Armenia; 4 Drug Discovery Lab, Department of Chemistry, City University of Hong Kong, 83 Tat Chee Avenue, Hong Kong 999077, China; 5 Department of Pediatric Oncology and Hematology, Yerevan State Medical University, 2 Koryun St, 0025 Yerevan, Armenia; 6 Armenian Pediatric Hematology and Oncology Group, 7 Nersisyan St., 0014 Yerevan, Armenia; 7 Department of Public Health, Yerevan State Medical University, 2 Koryun St, 0025 Yerevan, Armenia; 8 National Health Information Analytic Center, National Institute of Health, Ministry of Health of the Republic of Armenia, 49/4, Komitas ave, Yerevan, 375051, Armenia; 9 Department of Radiology, Armenian-American Wellness Center, 5 Heratsu Street, Yerevan 0025, Armenia; 10 Multiple Myeloma Program, National Cancer Institute, National Institutes of Health, 9000 Rockville Pike, Bethesda, MD 20892, USA

**Keywords:** Hodgkin lymphoma, incidence, Armenia, developing world

## Abstract

Hodgkin lymphoma (HL) accounts for roughly 10% of all lymphomas and 0.6% of all malignant tumours analysed worldwide yearly. Data regarding HL in developing world are exceptionally constrained. The main objective of this research is to investigate the incidence patterns of HL within the Republic of Armenia and to portray disease distribution according to age and sex. There is a very strict evidence on the frequency of HL in Armenia. The results of our research find out that the frequency of HL in Armenia has not changed altogether over the past 15 years and is comparable to that detailed from the USA and Europe.

## Introduction

Armenia is situated at the cross roads between Eastern Europe, Middle East and Western Asia occupying the north-eastern region of the historic Armenian plateau and highlands. As of 1 January 2018, the permanent population of Armenia was 2,972,732. The ethnic background of the population includes Armenians (98.1%), Yezidis, Russians, Assyrians, Ukrainians, Kurds, Greeks and other minorities.

Compared to non-Hodgkin lymphoma (non-HL), HL is a relatively infrequent lymphoma which is characterised by the pathognomonic malignant Reed–Sternberg cell with the cell of origin thought to be derived from the B-cell. Its annual incidence is 2–3 cases per 100,000 in Europe and the USA [1]. According to the Global Cancer Observatory (GLOBOCAN) (2018) [2], the greatest recorded all-age incidence rates of HL are in Greece, Lebanon and Saint Lucia. Rates are particularly low in India, Japan and China. Incidence rates of HL have not varied much since the mid-1970s, but mortality rates have steadily declined from 1.3 cases per 100,000 in 1975 to 0.3 cases per 100,000 in 2014. The 5-year overall survival (OS) for stage 1/2a is approximately 90%; on the other hand, stage 4 disease has a 5-year OS of approximately 60% [3–5].

There are numerous studies investigating the epidemiological aspects of HL in both developed and developing countries throughout the globe [2, 4, 6–8]. Cancer incidence and mortality vary significantly between developed and developing countries [4, 8, 9]. The variation in survival may be related to differences in the availability of resources (not limited to access to novel therapies but also technologies for diagnosis and monitoring) as well as other dimensions of access and quality of cancer treatment, early detection and prevention [8, 10, 11]. The lack of reliable statistics is a major concern in developing and under-developed countries including the Republic of Armenia. A synthesis of epidemiologic and molecular data from different clinical groups and diverse geographic regions might provide key information about the biology of this disease. Without health data on structure, treatment and outcome, it is difficult to determine whether healthcare implemented programmes and treatment protocols which have been successful in relatively wealth developed countries can be extrapolated to resource-limited settings. Therefore, population-based effectiveness studies in developing countries are essential in the development of their healthcare infrastructure [12]. For example, there is some early evidence suggesting that healthcare-related programmes and treatment protocols/procedures determined to be effective in the developed world might not always be as effective in the developing world which might be attributable to a combination of factors including patient, tumour, treatment and system-related factors [13–15]. In the developing world, the majority of patients are delayed in cancer diagnosis and present with advanced disease, which is more challenging to treat and cure, if even possible [16]. The delay in presentation is often due to a variety of factors, but commonly include a lack of awareness of the signs and symptoms of cancer, a lack of finances to travel to a hospital and cover the costs of diagnosis and treatment [17, 18].

There are few studies published from the developing world including Armenia regarding this topic. Fortunately, the success story in curing HL is not restricted to the developed world. Avagyan *et. al* [21] reported on a hospital based cohort study investigating the treatment challenges of HL in developing world [22]. This retrospective study showed that the treatment of HL could be successfully performed in a resource-limited setting and that the epidemiology and outcomes of HL in Armenia are possibly reaching those of developed countries [19]. The paucity of information and absence of a formal national cancer registry system in Armenia along the lines of Surveillance, Epidemiology, and End Results (SEER) in the USA, led us to conduct this study to evaluate the clinic-epidemiological characteristics of HL over a 15-year period.

## Methods and materials

In Armenia, the National Institute of Health collects information nationwide from the countries hospitals and outpatient clinics. We obtained population-based HL incidence data from the National Institute of Health of the Republic of Armenia which contains information on incidence for a period of 15 years (1 January 2000 to 30 December 2014) from all treatment centres across the country. Histologically proven HL (morphology and immunochemistry) cases, diagnosed by an oncologic pathologist, of all ages were included in the study. The incidence, age distribution and sex distribution were the parameters studied. Incidence rates were described as new cases per 100,000 person-years.

## Results

### Age-specific incidence

**Figure 1.**
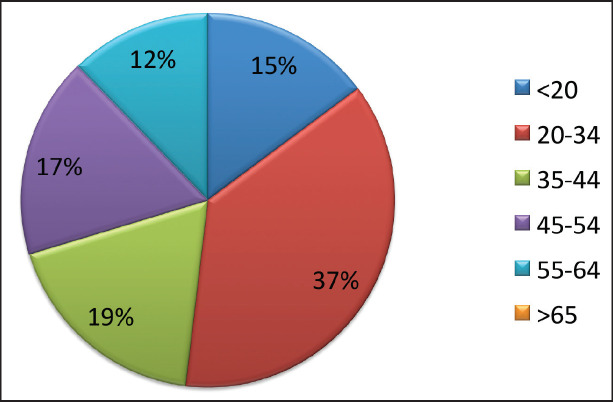
Age specific incidence of HL (%).

**Figure 2.**
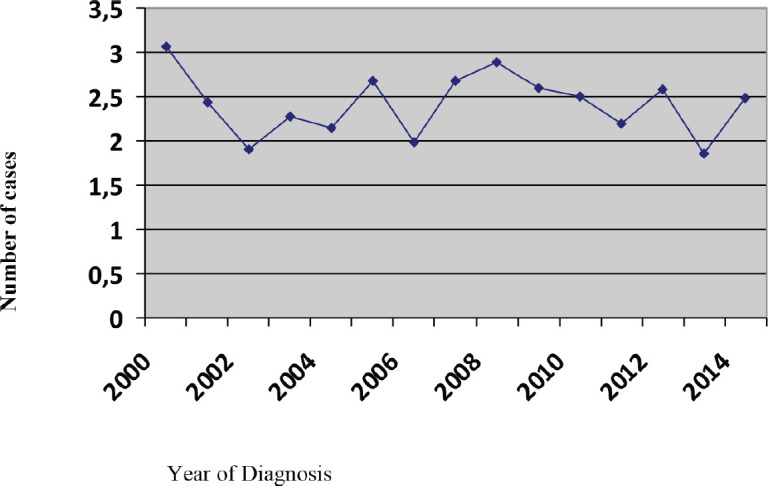
Incidence rate per 100,000 people.

During the 15-year period, 1,154 new cases of HL were diagnosed of which 148 (12.8%) patients were <20 years, 369 (32%) patients were 20–34 years, 183 (15.9%) patients were 35–44 years, 174 (15%) patients were 45–54 years, 122 (10.6%) patients were 55–64 years and 158 (13.7%) patients were ≥65 years of age (Figure 1; Table 1). In the majority of age groups, the highest morbidity rates were observed till 2007. In subsequent years, the rate has decreased and stabilised and is comparable to that observed in the USA and Europe. The peak incidence was different each year but it has bimodal age distribution. The average incidence per year during the 15-year period was 2.3 cases per 100,000 (Figure 2). The average age during the 15-year period was 38 (38.4 ± 17.8) per 100,000.

### Sex

In our patient cohort, there were 642 male patients compared to 512 females. The male to female ratio was 1.25:1 (Table 2). Age-specific incidence rates of HL were consistently higher among males than females in Armenia during the 15-year period. The largest sex-specific difference in age specific incidence rates occurred in the 0–20 and 20–34 year age groups. In the first group, male sex was more predominant and in the second group, female sex was more predominant. Relatively speaking, age-specific rates were generally 15%–30% higher in males than females.

## Discussion

Armenia’s health care system features three principal components: the national or ‘republican’ service level, which provides patients with large tertiary care hospital centres and an epidemiological service; the regional service level provides smaller hospitals and less sub-speciality care; the municipal and community service level provides primary health care services in community-based clinics with primary care physicians [23, 24].

Similar to many other developing countries, the Republic of Armenia also lacks a population-based national cancer registry (e.g. SEER) leading to assumptions being made as to the relative frequencies of the varying stages of different cancers based on global trends, which can be misleading and so efforts to improve cancer registries are required. Developing countries lack the funding to invest in comprehensive national cancer registries [25, 26]. In those developing countries where resources exist and cancer control policies have been formulated, there is often a lack of political will to implement these policies [27].

Current statistics are based largely on figures from hospital-based registries, which are often incomplete. Consequently, we had limitations regarding collection of some epidemiological data including data on patient survival. The major specialised medical institution dealing with the diagnosis and treatment of haematologic malignancies is the Hematology Center after prof. R.H.Yeolyan of the Ministry of Health located in the capital city Yerevan. The majority of HL patients are diagnosed and treated at this institution. A smaller volume of lymphoma patients receives their treatments at the National Center of Oncology named after V.A. Fanarjyan of the Ministry of Health, Muratsan Hospital Complex of Yerevan State Medical University. Lastly, there are a few private oncology clinics that also provide cancer treatment for those with financial resources, all the above centres are located in the same capital city. In the second and third largest cities, Gyumri and Vanadzor, respectively, each has a single state-owned outpatient clinic that also treats a few patients with HL.

Lymphomas are among the most common haematologic malignancies affecting the Armenian population. HL comprises a substantial proportion of these tumours [25]. In Armenia, HL is treated according to the standard international treatment regimens such as ABVD (doxorubicin, bleomycin, vinblastine and dacarbazine) and BEACOPP (bleomycin, etoposide, doxorubicin, cyclophosphamide, vincristine, procarbazine and prednisone) with or without radiation therapy [26–28]. The treatment of patients is primarily guided by the clinical stage of disease as determined by the Cotswolds classification [29]. Patients with early stage disease (stage I to II) are usually treated with a combination of chemotherapy plus radiation therapy. The amount of chemotherapy and dose of radiation differ for patients with favourable and unfavourable prognosis disease. Chemotherapy alone is an acceptable alternative for patients with favourable disease characteristics at higher risk for complications from radiotherapy. For patients with advanced stage (stage III to IV), combination chemotherapy is the main treatment. Radiation therapy may be used for select patients as consolidation [30, 31].

During the last few years, salvage therapy methods of treatment have been introduced at the Ministry of Health’s Hematological Center and Muratsan Hospital Complex of Yerevan State Medical University, which were not available in Armenia previously, including high-dose chemotherapy (HDC), stem cell harvest and rescue and a stem cell processing laboratory. HDC and autologous stem cell transplantation (ASCT) can salvage 40%–70% of patients with relapsed or refractory HL [32, 33]. More importantly, in resource limited countries without access to new therapeutics, HDC with stem cell rescue is much more financially feasible.

There are variations in the epidemiologic and clinico-pathological characteristics of HL in relation to geography and socioeconomic status. In the industrialised countries, HL has a bimodal incidence with the main peak in young adults of 15–35 years and the second one occurring after the age of 50. On the other hand, the disease appears more in young children in the developing countries. The current epidemiologic understanding of HL would suggest that the first peak (15–35 years) relates to specific factors affecting children (Epstein Bar Virus (EBV)-positive) and young adults (predominantly EBV-negative). The second peak (50 years) relates to the determinants of HL affecting older adults (largely resulting from a lack of immunologic control of latent EBV infection) [34, 35].

In one study from the USA covering the years from 2000 to 2007, 16,710 cases of HL showed that Asians and African Americans had a low incidence. The bimodal pattern of incidence also was less prominent for African American males. Asian and African American patients presented at a mean age of 38 years compared to 42 years for Caucasians (*p* < 0.001) [6]. Among the demographic characteristics of the different age groups, our results found a characteristic bimodal distribution, with the first, larger peak seen for adolescents and young adults (15–35 year age group) and a second, smaller peak occurring for adults (around 59 years of age).

The differences in incidence of HL by sex might suggest the importance of host factors [36–40]. In HL patients, high male to female ratio has been observed consistently in various studies [42]. A number of explanations have been put forward to explain this male predominant pattern of HL. Correa and O’Conor [41] suggested that females appeared to be less susceptible and more resistant than males to the initiating process and causative factors of HL. The excess of male patients in adult group might be partly explained on the basis of hormonal factors [39]. As expected, our results also demonstrated male predominance during this period of time. It should be noted that during the abovementioned 15 years, many significant changes took place in the diagnosis and treatment of haematologic malignancies in Armenia. Particularly, immunohistochemical analysis was launched in 2006, radiotherapy tech niques have changed significantly over the past decade, HDC and ASCT have become available and many doctors and nurses received training abroad from leading American and European institutions.

Unfortunately, there are also some gaps in managing cancer patients; for example, PET-CT isn’t available in Armenia and the only option for patients is to travel to another country to access this imaging modality, which in fact, for most patients is not an option due to financial reasons and the lack of more sensitive imaging modalities may very well impact patient outcomes [43–46]. Fortunately in the case of HL, the curative regimen of ABVD is relatively affordable, however, treating relapsed refractory HL with novel therapies such as immune-checkpoint inhibitors becomes financially problematic.

One of the greatest challenges of modern cancer medicine is to introduce evidence based curative procedures into developing countries to benefit patients of diverse socioeconomic classes [14, 19]. As a developing country, Armenia faces serious problems concerning diagnosis and treatment of these diseases. The reasons include late diagnosis, lack of treatment compliance, fragmented care, financial difficulties, cultural and social factors, distrust and belief in alternate medicine, etc. Another critical problem is the shortage of oncologists and specialised oncology nurses in Armenia [18, 19]. It is known, that experienced staff and a coordinated multidisciplinary approach are essential components of appropriate cancer care and result in significant cancer health disparities reduction. Avagyan *et al* [21] reported, that in the resource limited setting, it is possible to get similar results as those reported from developed countries by incorporating the appropriate standards of care and by the coordinated teamwork [47]. In Armenia, steps forward have already been implemented to make the treatment and follow-up of HL more efficient and complete. These include detailed collection of patient data and disease related characteristics, accurate follow-up and continuous training of staff. To the best of authors’ knowledge, the major limitation of this evaluation is that these parameters have not yet been validated in other population-based studies in Armenia.

## Conclusion

Armenia is an ethnically homogeneous country and any epidemiologic study becomes a population or cohort study, thus creating good prospects for conducting experimental and clinical trials. Retrospective studies like ours may provide baseline data for future prospective studies. Such data may also give an insight into the aetiology and pathogenesis of HL in Armenia’s geographical region. The results of our study show that the incidence of HL in Armenia has not changed significantly over the past 15 years and is similar to the rates in the western world.

## Conflicts of interest

The authors have no conflicts of interest to report.

## Funding statement

The authors received no financial support for the research, authorship, and/or publication of this article.

## Figures and Tables

**Table 1.** Incidence and distribution of HL in Armenia according to age.

	<20, *n*	20–34, *n*	35–44, *n*	45–54, *n*	55–64, *n*	≥65, *n*
2000	18	27	16	17	9	12
2001	14	17	20	10	9	8
2002	15	18	9	11	6	2
2003	5	20	15	15	7	11
2004	8	18	10	8	5	20
2005	6	31	10	8	12	19
2006	8	19	10	8	8	11
2007	9	36	10	15	7	11
2008	12	25	19	16	9	12
2009	12	22	14	16	12	9
2010	9	32	15	11	4	12
2011	7	31	10	8	12	19
2012	7	19	10	8	8	11
2013	5	36	10	15	7	11
2014	13	25	19	16	9	12

**Table 2.** Incidence and distribution of HL in Armenia according to year and sex.

Year	Male, *n*	Female, *n*	Male/female ratio
**2000**	53	46	1.15
**2001**	43	35	1.23
**2002**	31	30	1.03
**2003**	44	29	1.52
**2004**	34	35	0.97
**2005**	53	33	1.61
**2006**	37	27	1.37
**2007**	53	33	1.61
**2008**	48	45	1.07
**2009**	48	37	1.30
**2010**	45	38	1.18
**2011**	31	37	0.84
**2012**	38	40	0.95
**2013**	32	24	1.33
**2014**	52	23	2.26
**Total**	642	512	1.25

## References

[1] Perez-Callejo D, Zurutuza L, Royuela A (2018). Long-term follow up of Hodgkin lymphoma. Oncotarget.

[2] (2018). GLOBOCAN 2012: Estimated Cancer Incidence, Mortality and Prevalence Worldwide in 2012 v1.0.

[3] Howlader N, Noone AM, Krapcho M SEER Cancer Statistics Review (CSR) 1975–2016.

[4] Shenoy P, Maggioncalda A, Malik N (2011). Incidence patterns and outcomes for hodgkin lymphoma patients in the United States. Adv Hematol.

[5] Kaseb H, Babiker HM (2019). Cancer, Hodgkin Lymphoma.

[6] Hjalgrim H, Seow A, Rostgaard K (2008). Changing patterns of Hodgkin lymphoma incidence in Singapore. Int J Cancer.

[7] Kusminsky G, Abriata G, Forman D (2016). Hodgkin lymphoma burden in Central and South America. Cancer Epidemiol.

[8] Jemal A, Bray F, Center MM (2011). Global cancer statistics. CA Cancer J Clin.

[9] Fidler MM, Gupta S, Soerjomataram I (2017). Cancer incidence and mortality among young adults aged 20–39 years worldwide in 2012: a population-based study. Lancet Oncol.

[10] Bray F, Ferlay J, Soerjomataram I (2018). Global cancer statistics 2018: GLOBOCAN estimates of incidence and mortality worldwide for 36 cancers in 185 countries. CA Cancer J Clin.

[11] Torre LA, Siegel RL, Ward EM (2016). Global cancer incidence and mortality rates and trends--an update. Cancer Epidemiol Biomarkers Prev.

[12] Shamoon RP, Ali MD, Shabila NP (2018). Overview and outcome of Hodgkin’s lymphoma: experience of a single developing country’s oncology centre. PLoS One.

[13] Barrenho E, Miraldo M, Smith PC (2019). Does global drug innovation correspond to burden of disease? The neglected diseases in developed and developing countries. Health Econ.

[14] Magrath I, Litvak J (1993). Cancer in developing countries: opportunity and challenge. J Natl Cancer Inst.

[15] Price AJ, Ndom P, Atenguena E (2012). Cancer care challenges in developing countries: cancer challenges in Cameroon. Cancer.

[16] Connors JM I (2015). Hodgkin lymphoma: special challenges and solutions: Hodgkin lymphoma challenges and solutions. Hematol Oncol.

[17] Jaime-Pérez JC, Gamboa-Alonso CM, Padilla-Medina JR (2017). High frequency of primary refractory disease and low progression-free survival rate of Hodgkin’s lymphoma: a decade of experience in a Latin American center. Rev Bras Hematol Hemoter.

[18] Maddi RN, Linga VG, Iyer KK (2015). Clinical profile and outcome of adult Hodgkin lymphoma: experience from a tertiary care institution. Indian J Med Paediatr Oncol.

[19] Howell DA, Smith AG, Jack A (2013). Time-to-diagnosis and symptoms of myeloma, lymphomas and leukaemias: a report from the Haematological Malignancy Research Network. BMC Hematol.

[20] Safaryan L, Sargsyan L, Hakobyan L (2017). Diagnostic and therapeutic limitations and delayed diagnosis of pediatric hematologic malignancies in Armenia: a single-institution report. Clin Lymphoma Myeloma Leuk.

[21] Avagyan A, Danielyan S, Voskanyan A (2016). Treating adults with Hodgkin lymphoma in the developing world: a hospital-based cohort study from Armenia. Asian Pac J Cancer Prev.

[22] Biasoli I, Castro N, Delamain M (2018). Treatment outcomes for Hodgkin lymphoma: first report from the Brazilian Prospective Registry. Hematol Oncol.

[23] Richardson E (2013). Armenia: health system review. Health Syst Transit.

[24] Hovhannisyan SG (2004). Health care in Armenia. BMJ.

[25] Rastogi T, Hildesheim A, Sinha R (2004). Opportunities for cancer epidemiology in developing countries. Nat Rev Cancer.

[26] Valsecchi MG, Steliarova-Foucher E (2008). Cancer registration in developing countries: luxury or necessity?. Lancet Oncol.

[27] Stefan DC, Elzawawy AM, Khaled HM (2013). Developing cancer control plans in Africa: examples from five countries. Lancet Oncol.

[28] Daghbashyan SS, Melkikyan NA, Sahakyan LS (2016). Prevalence of hematological malignancies in Armenia. http://cttjournal.com/upload/iblock/675/6754f0d5d693bd4697ae5c95f0e703a0.pdf.

[29] Evens AM, Hutchings M, Diehl V (2008). Treatment of Hodgkin lymphoma: the past, present, and future. Nat Clin Pract Oncol.

[30] Rathore B, Kadin ME (2010). Hodgkin’s lymphoma therapy: past, present, and future. Expert Opin Pharmacother.

[31] Shanbhag S, Ambinder RF (2018). Hodgkin lymphoma: a review and update on recent progress: current progress in Hodgkin lymphoma. CA Cancer J Clin.

[32] Ultmann JE (1992). Classification of Hodgkin’s disease: yesterday, today and tomorrow. Eur J Cancer.

[33] Witkowska M, Majchrzak A, Smolewski P (2015). The role of radiotherapy in Hodgkin’s lymphoma: what has been achieved during the last 50 years?. Biomed Res Int.

[34] Yeoh KW, Mikhaeel NG (2011). Role of radiotherapy in modern treatment of Hodgkin’s lymphoma. Adv Hematol.

[35] Cocorocchio E, Peccatori F, Vanazzi A (2013). High-dose chemotherapy in relapsed or refractory Hodgkin lymphoma patients: a reappraisal of prognostic factors. Hematol Oncol.

[36] Akhtar S (2017). High dose chemotherapy and autologous stem cell transplantation in relapsed or refractory Hodgkin lymphoma: emerging questions, newer agents, and changing paradigm. Hematol Oncol Stem Cell Ther.

[37] Grotmol T, Bray F, Holte H (2011). Frailty modeling of the bimodal age-incidence of Hodgkin lymphoma in the Nordic countries. Cancer Epidemiol Biomarkers Prev.

[38] Sant M, Allemani C, Tereanu C (2010). Incidence of hematologic malignancies in Europe by morphologic subtype: results of the HAEMACARE project. Blood.

[39] Kılıçkap S, Barışta I, Ulger S (2013). Clinical features and prognostic factors of hodgkin’s lymphoma: a single center experience. Balkan Med J.

[40] Maggioncalda A, Malik N, Shenoy P (2011). Clinical, molecular, and environmental risk factors for hodgkin lymphoma. Adv Hematol.

[41] Correa P, O’Conor GT (1971). Epidemiologic patterns of Hodgkin’s disease. Int J Cancer.

[42] Cartwright RA, Gurney KA, Moorman AV (2002). Sex ratios and the risks of haematological malignancies: sex ratios and the risks of haematological malignancies. Br J Haematol.

[43] Englund A, Glimelius I, Rostgaard K (2018). Hodgkin lymphoma in children, adolescents and young adults – a comparative study of clinical presentation and treatment outcome. Acta Oncol.

[44] Berriolo-Riedinger A, Becker S, Casasnovas O (2018). Role of FDG PET-CT in the treatment management of Hodgkin lymphoma. Cancer Radiother.

[45] Kanoun S, Rossi C, Casasnovas O (2018). [18F]FDG-PET/CT in Hodgkin lymphoma: current usefulness and perspectives. Cancers (Basel).

[46] Subocz E, Hałka J, Dziuk M (2017). The role of FDG-PET in Hodgkin lymphoma. Contemp Oncol (Pozn).

[47] Keating NL, Landrum MB, Lamont EB (2013). Tumor boards and the quality of cancer care. J Natl Cancer Inst.

